# Universal testing in endometrial cancer in Sweden

**DOI:** 10.1186/s13053-024-00288-2

**Published:** 2024-08-22

**Authors:** Emil Andersson, Anne Keränen, Kristina Lagerstedt-Robinson, Sam Ghazi, Annika Lindblom, Emma Tham, Miriam Mints

**Affiliations:** 1 Department of Women’s and Children’s Health, Karolinska Institutet, Stockholm, Sweden; 2 Department of Molecular Medicine and Surgery, Karolinska Institutet, Stockholm, Sweden; 3 Department of Clinical Genetics and Genomics, Karolinska University Hospital, Stockholm, Sweden

**Keywords:** Endometrial cancer, Lynch syndrome, Universal testing

## Abstract

**Background:**

The aim of the study was to test a universal screening strategy on endometrial cancer to evaluate its effectiveness to find Lynch Syndrome (LS) cases to two established clinical criteria: Amsterdam II criteria, and the revised Bethesda criteria to select cases for prescreening with immunohistochemistry (IHC). Cases were subsequently screened for germline disease causing variants regarding the DNA mismatch repair (MMR) genes.

**Methods:**

IHC was performed on 221 endometrial cancer (EC) cases, using antibodies against the DNA mismatch repair proteins MLH1, PMS2, MSH2, and MSH6. MMR loss was found in 54 cases, and gene mutation screening was undertaken in 52 of those.

**Results:**

In this set of patients, the use of Amsterdam II criteria detected two (0.9%), the Bethesda criteria two (0.9%), and universal testing five (2.3%) cases of LS. The combination of universal testing and family history criteria resulted in detection of five patients (2.3%) with LS.

**Conclusions:**

Based on our results and other similar studies to date we propose a screening protocol for LS on EC tumors with prescreening using IHC for the four MMR proteins on all new EC cases diagnosed before 70 years of age, followed by mutation screening of all tumors with loss of MSH2 and/or MSH6 or only PMS2, plus consideration for mutation screening of all LS genes in cases fulfilling the clinical Amsterdam II criteria regardless of MMR status on IHC.

## Background

Endometrial cancer (EC) is the fourth most common cancer in women in the developed world and the incidence is rising due largely to the increase in obesity worldwide [1]. Although most endometrial cancers can be attributed to environmental factors, it is also known that inherited genetic factors account for a significant proportion of ECs. The most known genetic risk factor associated with EC is Lynch syndrome (LS). LS is caused by pathogenic variants in one of the four MMR genes *MLH1, MSH2, MSH6* and *PMS2* [2]. These genes code for the MMR complex that is responsible for correcting single nucleotide mismatches and small insertions/deletions during DNA-replication. A pathogenic variant in one of these genes causes a malfunction of the protein complex with inadequate DNA repair and will ultimately lead to an increased risk of oncogenic mutations for every replication cycle of DNA. LS is clinically characterized by an approximate 50% lifetime risk of being diagnosed with colorectal cancer (CRC) and EC, with half of the patients diagnosed with their first cancer before 50 years of age [3]. It is estimated that approximately 2% of all ECs [4] and 4% of all CRCs are caused by LS [5].

Previously, genetic screening for LS was solely based on clinical criteria. The Amsterdam criteria were designed to detect hereditary CRC but were later modified into the Amsterdam II criteria which also consider other LS-associated cancers. Although the Amsterdam II criteria have been shown to have a high specificity (98%) with high positive predictive value for identifying families with LS, the sensitivity of the criteria is poor (22%) [6]. Therefore, another set of clinical criteria called the revised Bethesda criteria were created to identify which CRC patients should be offered somatic testing for MMR defects [7]. Although Bethesda has a better sensitivity (82%), its specificity is lower (77%) subsequently leading to many genetic investigations with negative results [8]. Furthermore, the revised Bethesda are also created primarily for LS detection in CRCs. The clinical criteria also select for patients 50 years or younger while the fraction of LS associated cancers above 50 years of age have been shown to be as high as 24% of all LS-associated ECs [9] and in another study as high as 75% of all LS-associated ECs with 85% of them lacking any other previous cancer history [10]. These patients are likely to be missed by using clinical screening criteria.

Microsatellite instability (MSI) testing or immunohistochemistry (IHC) of MMR proteins has therefore started to be used as the primary screening method (so called universal testing), instead of clinical criteria for all newly diagnosed colorectal or endometrial cancers. This strategy has been shown to find twice as many carriers of a germline LS mutation.

One study from 2014 used MMR IHC and promotor methylation test to screen 381 randomly selected ECs and found 22 (5.8%) previously unknown LS carriers. Eight of these women were above 50 years of age and 10 lacked any family history of cancer [11]. The study showed that IHC as a primary screening tool is of importance for identifying individuals with LS. This in turn, will enable carrier individuals in the families to be included in screening protocols known to be of benefit for the long-term survival in the LS patients [12].

## Methods

### Aim

The aim of this study was to test a universal screening strategy on endometrial cancers diagnosed at Karolinska University Hospital between 2008–2012 and evaluate its effectiveness to find LS cases compared to the use of Amsterdam II and Bethesda criteria to select cases for prescreening with IHC and subsequent genetic screening of the MMR genes.

### Study population

The original RENDOCAS-cohort consisted of 481 endometrial cancer patients who had been diagnosed and treated at Karolinska Hospital, Stockholm, Sweden between 2008–2012 [13]. Tumor and blood samples had been collected and saved in the biobank from all participants. Of these cases there were in total 221 tumors available from the RENDOCAS-cohort with sufficient tumor material for IHC-staining on four separate slides necessary for this study. Pedigree data and personal cancer history was available for all patients. The Amsterdam II [14] and revised Bethesda criteria [15] (adapted to also include EC in the index patient) were used to categorize the families. Two of the 221 patients fulfilled the Amsterdam II criteria and 32 the revised Bethesda criteria.

### Immunohistochemistry (IHC)

Five µm sections of paraffin-embedded tumour samples were used. Paraffin was then dissolved in xylene, and the tissues were rehydrated by stepwise washing with ethanol (96% and 70% EtOH) in phosphate-buffered saline. Tissues were then immersed in a 2% solution of H_2_O_2_ in methanol at room temperature for 30 min to reduce background staining. Epitopes were retrieved through heating in citrate buffer (water bath, 96ºC for 15 min), and then the tumour samples were cooled at room temperature. The primary anti-MSH2, MSH6, MLH1 and PMS2 antibodies were diluted 1:200 in a blocking buffer (2% bovine serum albumin, 0.2% Tween-20, 10% glycerol, and 0.05% NaN3 in phosphate-buffered saline), were applied and left to stand for 60 min. Protein signals were visualised using the EnVision™ Detection Peroxidase/DAB system (Dako, Glostrup, Denmark). Nuclei were stained with Mayer’s hematoxylin (Dako). The samples were interpreted as MMR-deficient when displaying total or partial loss of expression in tumor nuclei. Stromal cells and lymphocytes in adjacent tissue served as an internal positive control. MMR proficient samples showed expression in tumor cells as well as in positive internal control cells [16].

Cases with heterogenous staining (variable staining in tumor nuclei) were reviewed by a second pathologist and regarded as an abnormal staining pattern indicating possible MMR deficiency. Faint staining in tumor cells or in internal positive control cells and cases with technical issues (poor fixation) were also reviewed by two pathologists. Technically failed slides were excluded. Clinical MMR IHC results were available for the two patients who fulfilled the Amsterdam II criteria, and those results were used in the final analysis.

### Germline sequencing

Mutation screening was performed with massive parallel sequencing (MPS) using the IonTorrent system (Applied Biosystems, Waltham, Massachussets, USA). In addition, a nested-PCR analysis of *PMS2* was performed as previously described [17]. For MPS, a custom-made design of common hereditary cancer genes was made using AmpliSeq designer (Applied Biosystems) and included all four MMR genes. Library preparation and sequencing were performed according to the manufacturer’s recommendations (Applied Biosystems). Output data was analyzed using the IonReporter software. The overall coverage was > 600x (150 Mb) per sample. Reported sequence variants from MPS were verified using Sanger sequencing. The following reference sequences were used: NM_000249.4 (*MLH1*), NM_000251.3 (*MSH2*), NM_000179.3 (*MSH6*), and NM_ 000535.7 (*PMS2*). The *PMS2* gene was also sequenced using Sanger sequencing as previously described (18), even though MPS had been performed.

In case no potential pathogenic sequence variant was detected using MPS, genomic DNA was also analyzed for the possibility of larger deletions/duplications in MMR genes using multiplex ligation-dependent probe amplification (MLPA) according to the manufacturer's instructions (assays P003, P008 and P072; MRC-Holland, Amsterdam, The Netherlands). Data was analyzed using GeneMarker software (SoftGenetics LCC, State College, PA, USA). Reported variants were classified according to ACMG-classification[18] and/or InSight VIC classification [19].

### Tumor methylation testing

For one case DNA extraction from FFPE tissue was performed on 10 µm thick sample slides using the Maxwell RSC DNA FFPE Kit AS1450 (Promega) according to manufacturer's protocol on a Maxwell RSC device from Promega. DNA was then analyzed in regard to promotor *MLH1* hypermethylation using methylation specific MLPA (assay ME011, MRC-Holland, Amsterdam, The Netherlands).

### Statistics

Descriptive analyses were used for this study.

## Results

Two of the 221 patients fulfilled the Amsterdam II criteria and 32 the revised Bethesda criteria. Among the 221 patients, Lynch syndrome was identified in two (0.9%) using the Amsterdam II criteria; in two (0.9%) using the Bethesda criteria; and universal testing resulted in five (2.3%) cases of verified LS. The combination of universal testing and family history criteria resulted in detection of five patients with LS (2.3%).

In total, 54 of 221 tumors (24.4%) had a loss of at least one MMR protein upon IHC. The mean age of EC diagnosis in this group was 68.3 years of age compared to 65.3 years in the group with normal MMR. In total, 45 of the 54 tumors (83.3%) had loss of MLH1 and PMS2, two (3.7%) had a loss of MSH2 and MSH6, and six (11.1%) had loss of only MSH6. One case had a loss of MLH1, PMS2 and MSH6: MSH2 was not assessable in this case (1.9%).

Genomic DNA was available and used for genetic screening on 52 of the 54 patients with MMR loss. 43 of 45 with loss of MLH1 and PMS2 on IHC had normal germline results. One patient had a germline pathogenic *MLH1* variant and fulfilled both Amsterdam II and our revised Bethesda criteria (patient 5 in Table 1). The remaining patient with loss of MLH1/PMS2 and normal MSH2/MSH6 staining had a pathogenic variant in the *MSH2* gene. This patient also fulfilled both Amsterdam II and the Bethesda criteria (patient 4 in Table 1). As an additional investigation on this patient we conducted a hypermethylation test on her tumor DNA that was positive for hypermethylation of the *MLH1* promotor.

**Table 1:** Patient characteristics with confirmed LS-mutations

	**Age of EC**	**Known LS-status?**	**AmsterdamII?**	**Bethesda?**	**IHC-result**	**Mutation**
Patient 1	62	No	No	No	**MSH6-**, MSH2? MLH1 + /PMS2 +	*MSH6*: c.3984_3987dup, p.(Leu1330Valfs*12)
Patient 2	57	No	No	No	**MSH6-,** MSH2? MLH1 + /PMS2 +	*MSH6* c.1910_1911del, p.(Leu637Profs*2)
Patient 3	50	No	No	No	**MSH6-,** MSH2? MLH1 + /PMS2 +	*MSH6* c.900dup, p.(Lys301Glufs*11)
Patient 4	65	Yes	Yes	Yes	**MLH1-/PMS2 -** MSH2 + , MSH6 +	*MSH2*: c.1786_1788del, p.(Asn596del)
Patient 5	58	Yes	Yes	Yes	**MLH1-/PMS2 -** MSH2 + /MSH6 +	*MLH1*: c.546-2A > G

*EC* Endometrial cancer, *LS* Lynch Syndrome, *IHC* immunohistochemistry, MMR-protein loss, +  = MMR-protein retained, ? = not assessable

Of the two patients with loss of both MSH2 and MSH6, one had a normal germline test result, and the other patient did not leave a blood sample to the study. The patient with a loss of MLH1, PMS2 and MSH6 had a normal germline test.

Of the remaining six patients with loss of only the MSH6 protein, three were found to have pathogenic germline variants in the *MSH6* gene (patients 1–3 in Table 1). One of these cases had a family history of a first degree relative with EC diagnosed < 50 years of age, but this was not sufficient to fulfill Amsterdam II or the revised Bethesda criteria (see Table 2). The other two had no known family history of LS-associated cancers. Three of the five LS patients were diagnosed before age 60 and two before age 70 (Table 1).

**Table 2:** Age distribution and patient characteristics of the whole data set

	**No of patients**	**LS pathogenic variant**
< 40 years	3	0
< 50 years	18	0
< 60 years	54	3
< 70 years	141	5
< 80 years	194	5
< 90 years	219	5
< 100 years	221	5
Amsterdam II	2	2
Bethesda	32	2
Bethesda only age	14	0
Bethesda age + other	4	0
Bethesda only other	14	2

EC-adapted Bethesda criteria: 1. Colorectal or uterine cancer diagnosed in a patient less than 50 years of age (age criteria), 2. Presence of synchronous, metachronous colorectal or other LS associated tumors regardless of age

3. Colorectal cancer with MSI-histology diagnosed in a patient who is less than 60 years of age

4. Colorectal cancer diagnosed in one or more first degree relatives with an LS-related tumor, with one of the cancers being diagnosed under age 50 years

5. Colorectal cancer diagnosed in two or more first or second degree relatives with LS-related tumors regardless of age

After IHC, 167 tumors (75.6%) were found to have intact MMR protein based upon on IHC. Of the tumors with intact MMR the mean age was 65.3 years at the time of EC diagnosis, and 25 patients (15.0%) fulfilled at least one Bethesda criteria, (Tables 2 and 3).

**Table 3:** Distribution of patients with positive EC-adapted Bethesda criteria in relation to MMR-status

Table 3	**Bethesda + **	**Bethesda -**
MMR loss	7 (13.0%)	47
MMR intact	25 (15.0%)	142

## Discussion

In total this study is comprised of 221 EC patients used to compare the detection rate of Lynch syndrome using different strategies. The use of Amsterdam II criteria had a detection rate of 0.9%, Bethesda criteria 0.9%, and universal testing 2.3%, which might be close to the LS frequency of LS in Sweden, even if the study used a relatively small number of cases.

A defect mismatch repair system or MSI phenotype can be screened for in tumor cells using PCR, next-generation sequencing techniques as well as IHC [20] to detect possible MMR deficiency. MSI and IHC usually display a high concordance [21], but both methods have their advantages as well as obstacles. MSI is a fast and non-user dependent technique, but cannot distinguish which specific MMR protein is involved in the MSI phenotype. It has also been shown that standard MSI-testing has a sensitivity of 80–90% for MLH1/PMS2 and MSH2 loss, but a reduced sensitivity of 55–77% to find deficient MMR caused by MSH6 loss [22]. IHC is dependent on a pathological evaluation, but can indicate which MMR-protein has been lost, and therefore helps select which gene should be screened in the germline DNA to confirm the LS diagnosis [23]. Furthermore, IHC has fairly equal sensitivity to both MLH1/PMS2 or MSH2/MSH6 loss with 83% sensitivity and 89% specificity regardless of gene involved (14).

However, one of our IHC findings were unexpected. One case with a MLH1/PMS1 loss on IHC did not have a *MLH1* mutation, but instead had a pathogenic germline variant in *MSH2*. The hypermethylation test later conducted on this patient’s tumor DNA turned out to be positive which indicates that her EC had a sporadic origin and was not caused by her inherited *MSH2* pathogenic variant. It should be noted that this patient fulfilled the Amsterdam II criteria, and other individuals in the family with the same pathogenic germline *MSH2* variant displayed loss of MSH2 and MSH6 upon IHC on their tumor tissue indicating that this variant does lead to protein loss in LS-associated tumors. Sporadic tumors can of course also occur in LS patients with increasing incidence in older ages. In fact, this patient was affected at 65 years of age and was the oldest in our LS cohort. If we would have used a study protocol with hypermethylation test on all MLH1/PMS2 losses before germline testing this patient would have been missed. However, with our strategy of performing germline screening of all four MMR genes on all tumors with IHC loss, we could still identify hereditary LS in this patient. Thus, our small study might suggest a benefit to always screen all four MMR genes when there is a MMR loss instead of only the gene suggested from the IHC result in cases that fulfill the Amsterdam II criteria.

44% of tumors with loss of MSH2 and MSH6 have a germline mutation in *MSH2,* but we only had available genomic DNA from one such patient with a normal germline test. Seven other cases had a loss of only MSH6 with three of them having germline mutations which is in line with other studies (23).

When using universal IHC on EC tumors as the first line screening tool our study found three previously unknown *MSH6* mutation carriers. None of these three patients fulfilled the Bethesda criteria, which is in line with previous literature on *MSH6* pathogenic variant carriers, who are known to have negative clinical criteria to a higher degree compared to *MLH1* and *MSH2* carriers, making them more difficult to find using clinical criteria as the primary screening method [24]. It is also in line with previous results that indicate that inherited *MSH6* mutations also make up a substantial part of all LS associated ECs [25]. One of these patients had a first degree relative with EC before 50 years of age which would increase suspicion of an inherited risk, but it was not sufficient to fulfill the revised Bethesda guidelines in their current design.

Our results are in concordance with other similar studies which have shown that universal IHC screening on endometrial and colorectal tumors would lead to approximately a doubled increase in detection of LS in randomly selected EC tumors or CRCs [26]. In November 2018 the American Cancer Society therefore updated their recommendations, and now suggest screening of all new diagnosed ECs with IHC to find MMR loss and which patients to continue investigation for LS [27].

Another important finding in our data is that Bethesda criteria did not show a high concordance with MMR protein loss (13.0%) and the patients with positive Bethesda were also evenly distributed between the group with loss of MMR and intact MMR (13.0% vs. 15.0%) (Table 3). This shows that Bethesda had a low sensitivity to detect MMR loss in EC and would probably perform poorly to detect LS in EC as the primary screening method. This can of course be explained by the fact that the Bethesda is designed primarily to detect LS in CRCs.

**Fig. 1:**
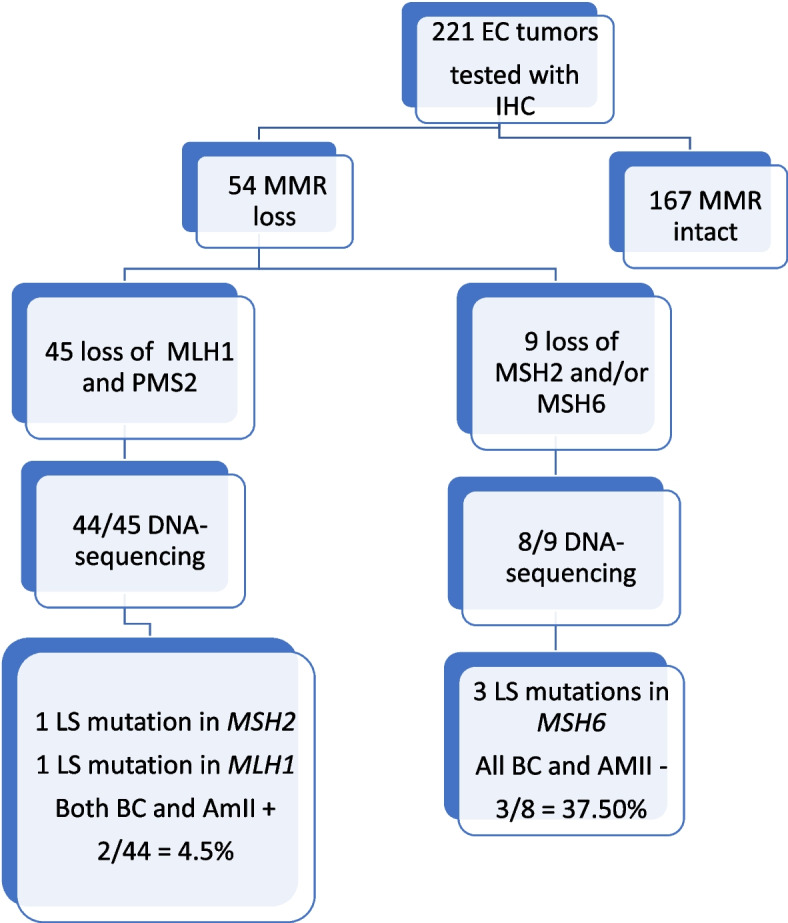
IHC and gene sequencing results. BC ±  = Bethesda criteria positive/negative, AmII ±  = Amsterdam II positive/negative

Although 25–30% of all EC tumors display the MSI phenotype or MMR loss, this is most often due to somatic mutations and not a result of inherited pathogenic variants (5). From the patients with MMR loss on IHC in our study the vast majority had a loss of MLH1 and PMS2 and only one showed a germline*MLH1* variant (Fig. 1). This finding is in line with a similar study who tested 500 EC tumors with IHC for MMR proteins and 132 turned out to have MMR loss on IHC with 100 of them with loss of MLH1 and/or PMS2 (75.8%). They further used *MLH1* promotor hypermethylation on these 100 MLH1 deficient tumors and found 83 of them (83.0%) to have positive hypermethylation. Interestingly the group with hypermethylated tumors were significantly older than those with no hypermethylation [28]. We did not conduct routine promotor methylation test in our study, but chose to screen for germline variants in these patients.

In future investigation protocols for LS it may be feasible to have a hypermethylation test done on MLH1 deficient tumors to exclude them for germline testing as long as those patients do not have a strong family history for LS which our *MSH2* carrier had. The high proportion of confirmed LS in patients with loss of MSH2 and/or MSH6 on IHC. (3/9, 33%,) is also in line with other studies on EC tumors. One study had 22 tumors in total with loss of either MSH2 and/or MSH6, twelve of which had a LS mutation (55%) and four additional cases had variants of unknown clinical significance (18%) [28]. A similar study to ours used IHC for MMR on 223 EC tumors in total and identified 60 with MMR-loss, nine of which had a MSH2 and/or MSH6 loss. In turn, seven of these nine cases underwent germline testing with four having a LS mutation (57%) [29].

It should be noted that 47 of 52 with loss of MMR on IHC that underwent genome screening did not have a germline disease causing variant in our study which indicates that the vast majority of MMR loss is explained by sporadic somatic mutations in the tumor. This could be an argument against universal IHC screening, but the MMR loss is still valuable information for the clinical management and outcome of EC. A study, that analyzed MMR loss and DNA methylation in 466 ECs found that MMR loss due to DNA methylation had a more advanced stage of cancer at diagnosis with worse clinical outcome than tumors with intact MMR or MMR loss without methylation [30]. The FDA has also approved the use of pembrolizumab as checkpoint inhibitor treatment in all tumors with MMR loss regardless of tumor origin [31].

This means that the MMR status will be important to know in EC from both a genetic and a clinical perspective, which further implies the use of universal screening with IHC on all EC tumors.

Of importance in the implementation of universal IHC screening on EC is the potential benefit of the patient, their relatives and the health care service of knowing if the person is a LS carrier in relation to the increased cost for tumor surveillance in the patient and their carrier relatives. Snowsill et al. [32] did a cost–benefit analysis on EC tumor screening from LS and compared three scenarios, where one option would be to not screen at all, one was to screen all ECs for a germline mutation directly, or to use IHC/MSI with or without methylation-test before proceeding to germline testing. They concluded that screening with IHC together with a methylation-test was the most cost- effective option of these three alternatives up to 65 years of age with a cost of 14.200 pound per quality-of-life year (QALY) gained. The study could not see any economic reason to screen patients older than 65 years due to the lower probability of LS associated cancer with increasing age, and the older age of the patient and her potential relatives. The obvious weakness with their model is that they did not compare the IHC and methylation alternative with a model based on clinical criteria for screening. Another study compared costs to screen CRCs universally or to have targeted IHC-screening based on age < 60 years and/or other Bethesda criteria. There was a 2.5 – sevenfold lower cost upon targeted screening compared to universal testing with the same detection rate of LS [33]. Although the detection rate is debatable, it can still be reasonable to use Bethesda criteria or age limits for IHC on a solely economic basis. The same group did a follow-up study with a cost benefit analysis for universal testing on all ECs. What they found was that IHC with methylation test is the most cost-effective option to diagnose LS in EC and the most cost-effective option for QALY gained if taking both the patient and relatives into account [34]. Kwon et al. showed that the most cost-effective strategy would be to screen with IHC on all ECs that have at least one first degree relative with a LS-cancer [35]. The other options were not cost effective at all, due to their low sensitivity for LS. The disadvantage with that analysis though is that they did not use methylation tests in their model, and the study is 10 years old when costs for IHC were higher. Still, there is a massive support for IHC as a primary screening tool with or without additional clinical criteria. Interestingly, their cost-effective strategy would have missed three LS-carriers we found in our study. A micro-cost effect study by Ryan et al. [36] confirmed the beneficial effect of adding methylation test together with IHC or MSI in the screening, where the costs were almost equal between using MSI or IHC.

The cost of MPS on tumor and germline DNA has dropped significantly in the last years but is not yet in clinical practice in Sweden. However, if the MPS technique continues to get cheaper, faster and easier to interpret, the next step in LS diagnostics might be paired tumor/normal MPS including MSI on all new EC cases.

## Conclusions

**Fig. 2:**
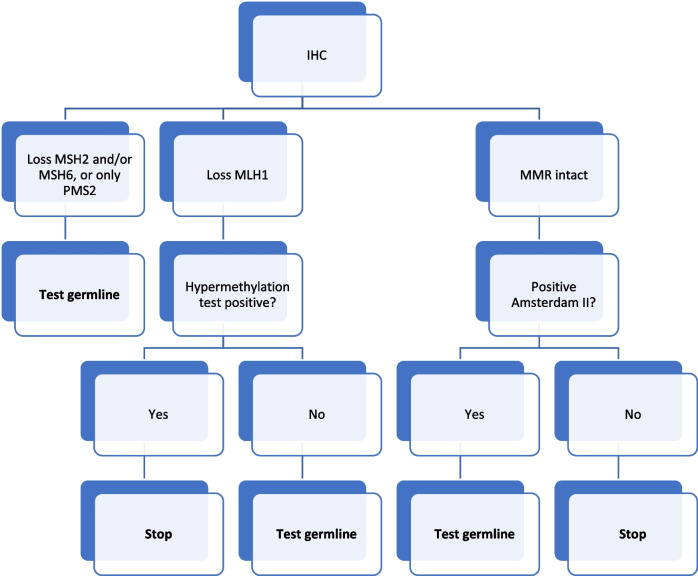
Proposed investigation for LS in newly diagnosed EC

Our study is one of the first in a Swedish EC cancer cohort to test universal screening with IHC for MMR loss. Based on our results and other similar studies to date we propose a universal screening protocol with IHC for MMR protein loss on all EC tumors diagnosed before 70 years of age for the purpose to increase the detection rate of LS. If the IHC shows loss of MLH1 we would consider to add a hypermethylation test before continuing with genomic screening. If IHC shows loss of MSH2 and/or MSH6 or only PMS2, germline testing of these genes is highly recommended due to the high frequency of LS in these patients (see Fig. 2). Furthermore, germline testing for all four LS genes should be considered for all families fulfilling the Amsterdam II criteria regardless of MMR status.

## Data Availability

The datasets used and/or analysed during the current study are available from the corresponding author on reasonable request.

## Abbreviations

EC: Endometrial cancer
IHC: Immunohistochemistry
BC: Bethesda criteria
AmII: Amsterdam II criteria
LS: Lynch Syndrome
MMR: mismatch-repair

## Data availability

No datasets were generated or analysed during the current study.
